# Micronutrients Dietary Supplementation Advices for Celiac Patients on Long-Term Gluten-Free Diet with Good Compliance: A Review

**DOI:** 10.3390/medicina55070337

**Published:** 2019-07-03

**Authors:** Mariangela Rondanelli, Milena A. Faliva, Clara Gasparri, Gabriella Peroni, Maurizio Naso, Giulia Picciotto, Antonella Riva, Mara Nichetti, Vittoria Infantino, Tariq A. Alalwan, Simone Perna

**Affiliations:** 1 IRCCS Mondino Foundation, 27100 Pavia, Italy; 2 Department of Public Health, Experimental and Forensic Medicine, University of Pavia, 27100 Pavia, Italy; 3 Endocrinology and Nutrition Unit, Azienda di Servizi alla Persona “Istituto Santa Margherita”, University of Pavia, 27100 Pavia, Italy; 4 Research and Development Unit, Indena, 20139 Milan, Italy; 5 University of Bari, Department of Biomedical Science and Human Oncology, 70121 Bari, Italy; 6 Department of Biology, College of Science, University of Bahrain, Sakhir Campus P. O. Box 32038, Bahrain

**Keywords:** celiac disease, vitamin B12, iron, folic acid, vitamin D, long-term GFD therapy (LTGFD), LTGFD with good compliance (LTGFDWGC)

## Abstract

*Background and objective*: Often micronutrient deficiencies cannot be detected when patient is already following a long-term gluten-free diet with good compliance (LTGFDWGC). The aim of this narrative review is to evaluate the most recent literature that considers blood micronutrient deficiencies in LTGFDWGC subjects, in order to prepare dietary supplementation advice (DSA). *Materials and methods*: A research strategy was planned on PubMed by defining the following keywords: celiac disease, vitamin B12, iron, folic acid, and vitamin D. *Results*: This review included 73 studies. The few studies on micronutrient circulating levels in long-term gluten-free diet (LTGFD) patients over 2 years with good compliance demonstrated that deficiency was detected in up to: 30% of subjects for vitamin B12 (DSA: 1000 mcg/day until level is normal, then 500 mcg), 40% for iron (325 mg/day), 20% for folic acid (1 mg/day for 3 months, followed by 400–800 mcg/day), 25% for vitamin D (1000 UI/day or more-based serum level or 50,000 UI/week if level is <20 ng/mL), 40% for zinc (25–40 mg/day), 3.6% of children for calcium (1000–1500 mg/day), 20% for magnesium (200–300 mg/day); no data is available in adults for magnesium. *Conclusions*: If integration with diet is not enough, starting with supplements may be the correct way, after evaluating the initial blood level to determine the right dosage of supplementation.

## 1. Introduction

Celiac disease (CD) is an immune-mediated systemic disorder triggered by the ingestion of gluten and prolamines in genetically predisposed individuals. It is characterized by inflammation of the small bowel mucosa—the immune reaction—which occurs after ingestion of gluten that leads to intestinal villous atrophy, crypt hyperplasia, and increased number of intraepithelial lymphocytes [1]. CD is a multifactorial disease and its pathogenesis involves both genetic and environmental factors [2]. Genetic composition for the development of the disease is evident. In fact, more than 90% of celiac patients are human leukocyte antigen (HLA)-DQ2 haplotype positive and almost all of the rest carry HLA-DQ8. These genes are necessary but not sufficient for CD development [3,4]. The predisposing DQ2 and DQ8 heterodimers are composed of the association of α and β chains. A recent meta-analysis showed that the HLA genotypes coding for DQ2 or DQ8 heterodimers, but also those including only the alleles of the respective β chains (regardless of the concomitant presence of DQ2 or DQ8 α chains) have an increased risk of developing pediatric CD [5]. Recently, another meta-analysis evaluated the predictive values of HLA-DQB1*02 allele, suggesting the major relevance of this specific allele, rather than the expression of the full DQ2 and/or DQ8 heterodimers, in raising the risk to develop pediatric CD [6]. In addition, a risk gradient according to single or double copy of HLA-DQB1*02 has been revealed [6]. Gluten ingestion represents the major environmental factor, contributing to the development of the pathology, but there are several other conditions involved in the etiology of CD, including viral infections, gut microbiota, breastfeeding, early life feeding practice, and smoking [3,4].

CD can occur at any stage of life and with a great variety of signs and symptoms. In fact, it is considered a multisystem immunological disorder rather than a disease restricted only to the gastrointestinal tract. Consequently, it is important to make diagnosis not only in individuals with classic gastrointestinal symptoms, but also in subjects who have a more nuanced or extra-intestinal clinical features, since the consequences can be important in both cases [2]. To date, nutritional therapy has been the only effective treatment for patients with CD that demands a strict compliance with a gluten-free diet (GFD). Non-adherence to the GFD increases the risk of morbidity and mortality, as a result of associated conditions, which include infertility, skeletal disorders and malignancy. Once diagnosed, patients should be tested for micronutrient deficiencies, including iron, folic acid, vitamin B12, and vitamin D [7].

The 2013 American College of Gastroenterology guidelines reported that micronutrient deficiencies (in particular iron, folic acid, vitamins B6 and B12, vitamin D, copper, and zinc) are frequent in celiac patients at the time of celiac diagnosis. Therefore, patients with newly diagnosed celiac disease, micronutrient deficiencies should be found and integrated. These tests should include iron, folic acid, vitamin D, vitamin B12 and more [7].

Following the United Kingdom 2015 National Institute for Health and Care Excellence guidelines, it was reported that some patients with celiac disease may need additional nutritional supplements, mainly in the early stages after diagnosis, suggesting, however, that this should be identified through an appropriate ongoing monitoring and that integration should begin after a full evaluation [8].

These two guidelines are derived from, and in agreement with, the more recent reviews demonstrating that in celiac patients, at time of diagnosis, nutritional deficiencies are often found in vitamins and minerals, such as folic acid, vitamin B12, vitamin D, calcium, magnesium and zinc.

However, at the same time, in subjects undergoing GFD for a long time with good compliance, it has been described that micronutrient deficiencies may persist due to an inadequate full reintegration of the mucous membrane [9]. Some patients with long-term treated CD may still have abnormal small bowel mucosa and persistent villous atrophy on follow-up, with or without ongoing or recurrent symptoms, despite an apparently GFD [4,10]. According to Lanzini et al., the complete recovery of duodenal mucosa with histological normalization, after a median 16 months GFD in patients diagnosed at an adult age occurs only in 8% of cases [11]. The majority of adult patients achieving remission with intraepithelial lymphocytosis (65%) and a substantial proportion showing no-change (26%) or deterioration (1%) of duodenal histology [11]. This condition seems to be more common in adults older than an age of 50 years [12], but occurs even in 19% of children who underwent follow-up biopsy at least 1 year after starting the GFD [13]. When other possible causes of villous atrophy are excluded, refractory celiac disease is diagnosed [4]. Even in the absence of symptoms this condition is not positive, because it may predispose to severe complications, such as osteoporosis and malignancy [14].

Moreover, gluten-free products are usually low in some micronutrients, such as magnesium and folic acid, and gluten-free cereals found in nature have a lower magnesium content compared with gluten-containing ones [9].

This topic is highly debated in the literature. In fact, there is a widespread agreement on the importance of supplementation at the time of diagnosis, but there is still no consensus for when and what additional nutrients are needed in subjects on long-term GFD (LTGFD).

Given this background, the aim of this narrative review is to evaluate the literature that considers blood nutritional deficiencies in celiac subjects on LTGFD therapy with good compliance (LTGFDWGC) in order to prepare dietary supplementation advice for these patients.

## 2. Materials and Methods

The present narrative review was performed following the steps by Egger et al. [15] as follows:

1. Configuration of a working group: three operators skilled in clinical nutrition (one acting as a methodological operator and two participating as clinical operators).
2. Formulation of the revision question on the basis of considerations made in the abstract: “the state of the art on nutritional deficiencies in celiac subjects on LTGFD therapy with good compliance; “good compliance” was defined as those patients who had been apparently carefully compliant with the GFD for a at least one year based on dietary history, and this was supported by the absence of coeliac antibodies (if present at diagnosis), or having a healed duodenal biopsy if previous coeliac serology was unavailable”.
3. Identification of relevant studies: a research strategy was planned on PubMed (Public MedIine run by the National Center of Biotechnology Information (NCBI) of the National Library of Medicine of Bathesda (USA)) as follows: (a) Definition of the keywords (celiac disease; vitamin B12; iron; folic acid; vitamin D; calcium; zinc; magnesium; LTGFD therapy; LTGFDWGC), allowing the definition of the interest field of the documents to be searched, grouped in quotation marks (“…”) and used separately or in combination; (b) use of: the Boolean (a data type with only two possible values: true or false) AND operator, that allows the establishments of logical relations among concepts; (c) Research modalities: advanced search; (d) Limits: time limits: papers published in the last 20 years; humans; adults; languages: English; (e) Manual search performed by the senior researchers experienced in clinical nutrition through the revision of reviews and individual articles on management of inflammation and oxidative stress by dietary approach in celiac patients published in journals qualified in the Index Medicus.
4. Analysis and presentation of the outcomes: we create paragraphs about different micronutrients, and the data extrapolated from the “revised studies” were collocated in tables; in particular, for each study we specified the author and year of publication and study characteristics.
5. The analysis was carried out in the form of a narrative review of the reports. At the beginning of each section, the keywords considered and the type of studies chosen are reported. We evaluated, as is suitable for the narrative review, studies of any design which considered the nutritional deficiencies in celiac adult subjects on LTGFD therapy with good compliance.

## 3. Results

**Figure 1:**
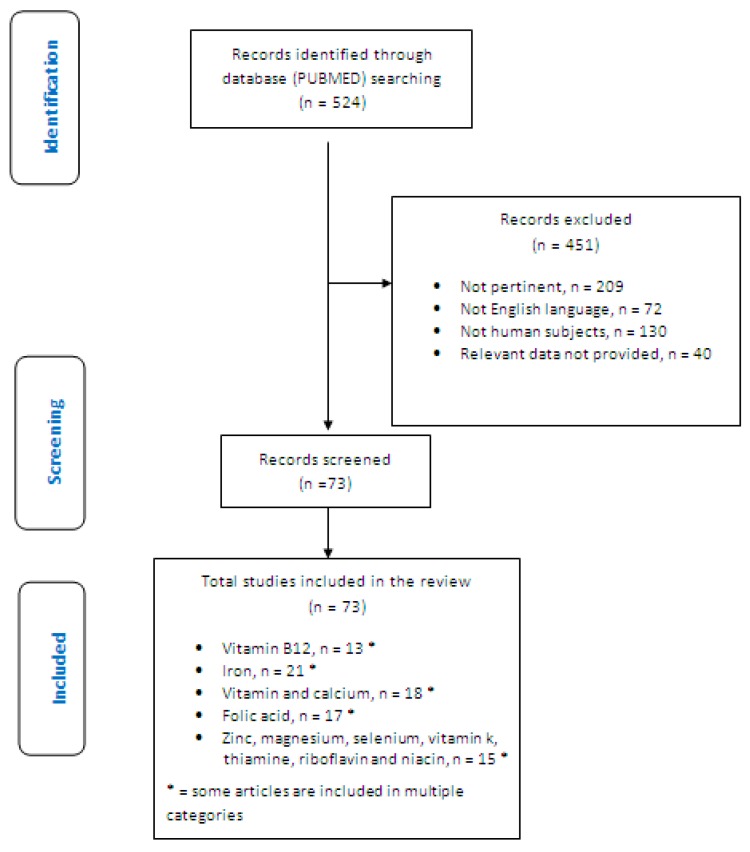
Flowchart of the study.

This review included 73 eligible studies and the dedicated flowchart is shown in Figure 1.

Table 1 shows the reviews made on nutrient deficiencies in celiac patients at time of diagnosis and after LTGFDWGC.

Table S1 shows the studies concerning circulating levels and supplementation of micronutrients in celiac patients after LTGFDWGC.

The literature shows that nutritional deficiencies, considered by evaluating the blood values of these micronutrients, in celiac subjects on LTGFD with good compliance, relate to vitamin B12, folic acid, vitamin D, calcium, iron, magnesium, zinc, selenium, thiamine, riboflavin, niacin and vitamin K (Table 1).

### 3.1. Vitamin B12

This research was carried out based on the keywords “vitamin B12” AND “supplementation” AND “long-term GFD with good compliance” AND “celiac patient” OR “celiac disease”. Of the 13 studies that were taken into account, 6 were review-type papers, 3 were prospective studies, 2 were observational studies and 2 were randomized controlled trials.

The absorption of dietary vitamin B12 occurs mainly in the terminal ileum through an active, specific and saturable transport mechanism. Vitamin B12 is released from food proteins after exposure to gastric acid. Vitamin B12 links to a salivary and gastric R protein; then pancreatic proteases destroy the R protein in the duodenum, releasing cobalamin which creates a complex with intrinsic factor (IF) that is secreted by the parietal cells in the stomach. The complex B12-FI migrates up to the terminal ileum aided by intestinal peristalsis, and binds itself through its proteic fraction to a specific cellular receptor. The complex dissociates and cobalamin enters the enterocytes of the small intestine. When the vitamin is administered orally in high doses, a small proportion along the entire intestine is absorbed through a passive diffusion mechanism.

Absorption site remains relatively preserved in patients with CD, so deficiency of vitamin B12 should be unusual. Nevertheless, numerous studies have shown that circulating levels of this vitamin are inadequate in about 5–40% of patients with CD at diagnosis [19,20,21,22] and in about 2.9-41% of patients following a GFD [20,21].

A real link exists between CD and vitamin B12 deficiency, but it has not been established. Some studies have shown that GFD and, where required, supplementation with vitamin B12 is effective in resolving neurological complications associated with deficiency of this vitamin. It has been shown that concentration of vitamin B12 tends to normalize in patients with a LTGFD [17,23].

However, there is evidence that supplementation may also be useful in subjects undergoing GFD. Hallert et al. [24] conducted a double-blind study to evaluate the effects of supplementation with B vitamins in adult CD patients for a long time, which involved daily administration of 0.8 mg of folic acid, 0.5 mg of cyanocobalamin, and 3 mg of pyridoxine for a period of 6 months. In these patients, there was improvement of psychiatric symptoms, and a significant return to normal vitamin B12 values with reduction of homocysteine values, which is often elevated in patients with vitamin B12 deficiency. Indeed, the catabolism of homocysteine requires vitamin B12 and folate. Consequently, hyperhomocysteinemia may reflect a deficit of both nutrients [25]. Great attention to the levels of homocysteine is needed in patients with CD. Celiac patients appear to have an increased risk of venous thromboembolism and vascular disorders [26] and high levels of homocysteine is a risk factor for these chronic diseases [27]. Supplementation of vitamin B12 and folate tends to decrease homocysteine values [24], so it could represent a prevention behavior.

In some patients, it is therefore necessary to integrate this element, even when following a strict GFD. In such cases, administration of vitamin B12 can be given via the oral or intramuscular routes.

In a study carried out by Bolaman et al. on general populations with megaloblastic anemia due to deficiency of cobalamin, oral supplementation was as effective as intramuscular treatment. Oral administration seems to be less costly and more tolerable than intramuscular delivery [28]. Furthermore, a review carried out by Vidal-Alaball et al. on general populations showed that in patients with a deficiency of vitamin B12, oral administration of 2000 mcg/day or 1000 mcg/day, followed by 1000 mcg/week and then 1000 mcg/month, can be as effective as intramuscular administration in showing improvement in hematological and neurological levels [29].

In patients with CD, supplementation is recommended for those in which there remains a blood deficiency despite GFD. Hallert et al. have shown how the oral administration of 500 mcg of cyanocobalamin in subjects undergoing GFD is effective in restoring the homocysteine value (which is an indirect measurement of vitamin B12 and folate). It suggests that the absorption after oral administration, especially in subjects undergoing GFD, is effective [24]. Furthermore, Theethira et al. suggested measuring vitamin B12 levels at diagnosis and then every 1–2 years for symptoms, and to treat with 1000 mcg orally until levels normalize, and then considering daily gluten-free multi vitamin/mineral supplementation [30].

In conclusion, considering the site of absorption (terminal ileum) of vitamin B12, which remains relatively preserved in patients with CD, deficiency of this vitamin should be infrequent; however, circulating levels of this vitamin could remain inadequate up to 41% in LTGGFDWGC patients. Given this background, in addition to its pivotal role in preventing hyperomocysteinemia, an annual routine follow-up of blood vitamin B12 level is mandatory in subjects undergoing LTGFD. Regarding dose and route of administration, the literature showed that in celiac patients with vitamin B12 deficiency, oral administration of 1000 mcg of vitamin B12 until levels normalized, followed by daily gluten-free multi-vitamin/mineral supplementation with 500 mcg of vitamin B12 is effective [30].

### 3.2. Iron

This research was carried out based on the keywords “iron” AND “supplementation” AND “long-term GFD with good compliance” AND “celiac patient” OR “celiac disease”. Of the 21 studies that were taken into consideration, 8 were prospective studies, 5 were reviews, 3 were observational case studies, 2 were case control studies, 1 was a randomized controlled trial, 1 was a report and 1 was a guidelines.

Iron deficiency often occurs in celiac patients, and it is followed in many cases by iron deficiency anemia. Studies have shown that the prevalence of this event in patients with newly diagnosed CD seems to be between 10–80%. The prevalence of deficiency after 6 months of GFD is about 70%, after 1 year, it is about 50%, and after 2 years, it is about 40% [17,19,31,32,33,34,35,36,37].

Iron is an essential trace element, being part of the heme structure, the non-protein component of numerous iron proteins (such as hemoglobin, myoglobin and cytochromes). Its excretion cannot be controlled, so the amount of iron in the body depends mainly on its absorption, which takes place in the duodenum and proximal jejunum.

Iron deficiency anemia in CD patients mainly arises from malabsorption, although the possibility of intestinal bleeding cannot be excluded and must be considered [38,39].

In the general population, initial treatment of iron deficiency should be continued until hemoglobin and iron stores are normalized. This goal is usually obtained with oral iron administration. Although it is occasionally recommended to take iron supplements before breakfast in order to increase the absorption, this significantly reduces the tolerance. For this reason, it seems reasonable to suggest the administration with food. All ferrous salts, including ferrous fumarate, ferrous lactate, ferrous succinate, ferrous glutamate, and ferrous sulphate, share similar bioavailability. Preparations of iron glycinate represent a valid therapeutic alternative, since they have a good bioavailability and a lower frequency of side effects, such as constipation [40,41,42].

Treatment with oral iron is, in the general population, slow in reaching its goal, and good compliance is required to be successful. In addition to this, anemia is often severe, and a quick response is necessary. Sometimes the tolerance is poor, and in these situations the use of parenteral iron is fully justified. The efficacy and safety of parenteral iron sucrose use have been demonstrated in several clinical studies and have been confirmed by extensive clinical practice [43]. To supply the quantities required, several doses are needed. Other intravenous drugs such as ferric carboxymaltose have been introduced [44] and would require fewer infusions to provide the required dose. Ferric carboxymaltose is a robust and stable non-dextran intravenous iron formulation with the advantage of having a very low immunogenic potential, and therefore is not predisposed to anaphylactic reactions. Its properties permit the administration of large doses (15 mg/kg; maximum of 1000 mg/infusion) in a single and rapid session (15-min infusion) [45].

Therapy on the general population should start with a low dose and the intake should be constant until the iron deposits are not restored [40]. It is fundamental that treatment of an underlying cause should prevent further iron loss, but all patients should have iron supplementation, both to correct anemia and to replenish body stores. This is achieved most simply and cheaply with oral ferrous sulphate 200 mg twice daily. Lower doses may be as effective and better tolerated, and should be considered in patients not tolerating traditional doses. Other iron compounds (e.g., ferrous fumarate, ferrous gluconate) or formulations (iron suspensions) may also be tolerated better than ferrous sulphate. Oral iron should be continued for 3 months after the iron deficiency has been corrected so that stores are replenished [46].

Regarding celiac patients, in subjects in which iron supplementation is needed, it should be started orally. In the decision on when to start, some authors suggest undertaking supplementation in the moment in which the intestinal lesions are healed [40], while other studies suggest taking the supplement immediately at the time of diagnosis, without waiting for the healing of the mucosa [47]. In most of these patients, GFD is enough to solve the framework of anemia [48], although it may take a long time. In other cases it is necessary to help the patient with supplementation [36]. A study carried out on 25 pediatric patients with CD and iron deficiency showed good efficacy of oral administration of iron, (investigated with ferrous bisglycinate chelate 0.5 mg per kg body weight, reaching a maximum of 28 mg) both in patients with GFD and in those newly diagnosed [47]. A study carried out on celiac pediatric patients showed that the therapeutic dose in pediatric patients with an iron deficiency is 3 mg of elemental iron per kg body weight per day. The prophylactic dose in pediatric patients is 2 mg of elemental iron per kg body weight per day reaching a maximum dosage of 30 mg per day [36].

Since gluten-free products are characterized by a low iron content [49], the intake of foods rich in this mineral, such as meat, should be recommended to patients initiating a GFD.

Theethira et al. suggested measuring serum iron and ferritin at diagnosis, repeating every 3–6 months until ferritin was normal, and then every 1–2 years for symptoms. Moreover, they suggested iron supplements (325 mg), 1–3 tablets based on initial ferritin level until iron stores are restored, and consideration of intravenous (IV) iron for severe symptomatic iron deficiency anemia or intolerance of oral iron.

In conclusion, it seems that when long GFD, including food tips to consume adequate dietary iron, is not enough to restore iron levels (40% of LTGFDWGC subjects are iron-deficient), the right approach could be to start with oral administration of iron.

Based on this background, a semi-annual steady and routine follow-up of blood iron and ferritin levels is mandatory in subjects undergoing LTGFD [30].

### 3.3. Folic Acid

This research was carried out based on the keywords “folic acid” AND “supplementation” AND “long-term GFD with good compliance” AND “celiac patient” OR “celiac disease”. A total of 17 studies were taken into consideration. Among these studies, 7 were prospective studies, 5 were review studies, 2 were observational case studies, 2 were randomized controlled trials, and 1 was a report.

The term “folate” describes the vitamer group B, based on the main structure of folic acid, which shares the same vitamin activity. This group of vitamins is essential for the synthesis and repairing of DNA, and they also act as cofactors for the enzymes involved in several biological reactions. Folate occurs naturally in some foods, and its synthetic form, folic acid, is added to many food products to increase the dietary intake. An example of supplementation is the addition of folic acid to wheat flour, which has been introduced in 52 countries worldwide since 2007 [50].

Folate deficiency was detected in about 10–85% of adult patients with CD at diagnosis, and in about 0-20% of patients following a GFD [17,20,22,51,52,53,54]. Usually the folate deficiency in CD occurs in patients with lesions in the ileum [48,55,56].

Tighe et al. compared in the general population the effectiveness of 0.2 mg folic acid per day with that of 0.4 and 0.8 mg/day in lowering homocysteine concentrations over a 6-month period. It has been seen that folic acid significantly reduces the risk of stroke overall by 18%, but to a greater extent by up to 25% in those trials that showed greater homocysteine lowering or in persons with no history of stroke. The lowest dose of folic acid required to achieve effective reductions in homocysteine is controversial but important for food fortification policy given recent concerns about the potential adverse effects of overexposure to this vitamin. This study supports the potential benefit of enhancing folate status and/or lowering homocysteine in the primary prevention of stroke. The authors suggest that a folic acid dose as low as 0.2 mg per day can, if administered for 6 months, effectively lower homocysteine concentrations [57].

Numerous studies have shown that a GFD would be sufficient to normalize folate status [21,48,58], but one other [23] show that in a certain percentage of patients, folate levels remain low despite GFD maintained for over 10 years. A possible explanation for this phenomenon is the reduced content of folate in gluten-free foods as previously described by Thompson [49]. Another hypothesis is that in celiac patients genetic alterations of the proteins involved in absorption and metabolism of folate may be present [17].

Hallert et al. suggested providing patients with good information about folate-rich foods. They also recommended starting supplementation in patients who show blood deficiencies after GFD [17,23].

Dosage should be decided in relation to the initial value of the subject. In a study conducted by Hallert et al. on celiac patients, they administered 0.8 mg of folic acid leading to normalization of homocysteine values [24].

In conclusion, folate deficiency was detected in up to 20% of patients on LTGFDWGC. Given this background, a semi-annual routine follow-up of blood folic acid level is mandatory in these subjects. Dosage of supplementation should be decided in relation to the detected value in the subject. The literature suggests supplementation with 1 mg/day of folic acid for 3 months, followed by a reduction to 400–800 mcg/day [30] or with 0.8 mg of folic acid [24] in order to improve the poor folate status.

### 3.4. Vitamin D and Calcium

This research was carried out based on the keywords “vitamin D” OR “calcium” AND “long-term GFD with good compliance” AND “supplementation” AND “celiac patient” OR “celiac disease”. A total of 18 studies were taken into account. Of these studies, 5 were review studies, 4 were prospective studies, 3 were case reports, 2 were observational studies, 2 were guidelines, 1 was a case-control study and 1 was a meta-analysis study.

#### 3.4.1. Vitamin D

The cholecalciferol, or vitamin D3, can be synthesized in the basal layers of the epidermis starting from cholesterol, by the action of ultraviolet rays of sunlight, and this should be the main source of vitamin D for the body. Another source of vitamin D is food, from which the absorption occurs mainly in the terminal ileum.

Numerous studies have shown low levels of vitamin D in many untreated celiac patients. Vitamin D deficiency, investigated through blood value, was detected in about 8–88% of adult CD patients at diagnosis, and in about 0–25% of patients following a GFD [17].

Nevertheless, certain patients, mainly post-menopausal women, continue to present bone density levels below the normal range [59,60]. This seems to be partly due to lack of vitamin D1.

If GFD is not sufficient to bring the values in the normal range, supplementation of vitamin D and calcium is required [17,61,62,63]. The Endocrine Society guidelines recommend, for the general population, that serum levels of vitamin D are at least equal to 30 ng/mL, and that it is necessary to decide the dosage of supplementation in relation to the initial value of the subject [64].

A meta-analysis conducted on general populations by Shab-Bidar et al. shows that a significant increase in serum levels of vitamin D in adults is achieved with a dose of ≥800 UI/day, at least after 6–12 months of supplementation [65].

Consider that for the celiac patient, a commonly applied strategy in cases of serious vitamin D deficiency is to prescribe a “loading dose” (e.g., 50,000 UI/week for 8 weeks) followed by reduced doses, as shown by Duerksen in a case report study of a woman with CD [66].

In a study aimed at detecting the effects of calcium and vitamin D supplementation in celiac children by Muzzo et al., daily supplementation with 1000 mg of calcium and 400 UI of vitamin D for 24 months was shown to have beneficial effects on the bone mass of celiac patients in whole body and femoral neck measurements; however, these values did not reach the controls [67].

A recent study carried out by Zanchetta et al. suggests an intake of 1000–1500 mg/day of calcium in two or more divided intakes of dairy products and a dose of vitamin D necessary to maintain a blood level of 30 ng/mL [68].

Moreover, Theethira et al. suggested measuring vitamin D levels at diagnosis, repeating every 3 months until levels are normalized, and then every 1–2 years or for symptoms. If necessary, integrate with 1000 (or more-based serum level) UI/day or 50.000 UI weekly if level is <20 ng/mL.

In conclusion, blood vitamin D deficiency was detected in about 0–25% of patients following a LTGFDWGC [69].

#### 3.4.2. Calcium

Calcium deficiency was detected in about 41% of adult patients with CD at the diagnosis [32] and 3.6% of treated children [70]. This seems to be due to malabsorption related to intestinal epithelial damage, but it could also be linked to a reduced expression of a protein regulated by vitamin D that controls the absorption of calcium [17,71]. Calcium absorption is impaired due to mucosal atrophy. Therefore, to avoid hypocalcemia, parathyroid hormone increases substantially (secondary hyperparathyroidism) and stimulates osteoclast-mediated bone degradation. Calcium is then obtained from the skeleton reservoir, but this high remodeling state can lead to osteopenia and osteoporosis, altering bone microstructure and increasing fracture risk [68].

In a study relating to the persistence of calcium deficiency despite a GFD, Kavak et al. undertook the analysis of reduced intake and absorption, rather than the percentage of shortage investigated by blood values in children patients after GFD [70]. The authors reported a reduction in calcium intake in about 76–88% of patients adhering to a GFD [69]. Pazianas et al. described a reduced fractional calcium absorption in adult celiac patients adhering to a GFD despite adequate calcium intake. Taken into account their reduced fractional calcium absorption, the authors concluded that their daily dose should be at least 1200 mg per day [72]. Larussa et al. showed that asymptomatic patients following a GFD for at least 2 years showed normal circulating serum calcium and parathyroid hormone (PTH) levels [73,74]. Zanchetta et al. showed a significant reduction of bone resorption parameters and PTH values, with a significant increase in serum calcium and vitamin D after GFD. Sategna-Guidetti et al. described significant improvement of bone mineral density values in newly diagnosed CD patients after 1 year of following a GFD [53,68].

Regarding supplementation, Zanchetta et al. suggested 1000–1500 mg/day in two or more divided intakes of dairy products. The authors concluded that calcium supplementation may be an option if the patient is not able or willing to fulfill the required intake through dietary means [68]. Theethira et al. found that more than 50% of patients consume less than the recommended daily intake of calcium. They recommended that CD patients undergo regular assessments with a dietitian and that the recommended intake of calcium, including supplementation should be 1200–1500 mg/day [30].

### 3.5. Other Micronutrients

This research was carried out based on the keywords “zinc” OR “magnesium” OR “selenium” OR “vitamin K” OR “thiamine” OR “riboflavin” OR “niacin” AND “supplementation” AND “long-term GFD with good compliance” AND “celiac patient” OR “celiac disease”. Of the 15 studies that were taken into account, 9 were review studies, 2 were report studies, 2 were prospective studies, 1 was an observational study, and 1 was a case-control study.

#### 3.5.1. Zinc

Zinc deficiency was detected in more than 50% of adult patients with CD at diagnosis, and between 0–40% of patients following a GFD [17]. The lack of this mineral seems to be linked in part to the its reduced absorption, due also to the degree of inflammation of the mucosa.

In the review by Theethira et al., they proposed to measure the serum zinc levels of CD patients at diagnosis and repeat after 3 months until the level is normal, followed by every 1–2 years for symptoms. They also suggested zinc supplementation between 25–40 mg/day until zinc levels were normal [30].

#### 3.5.2. Magnesium

Magnesium deficiency was detected in about 21.4% of adult patients with CD at the diagnosis, and a similar percentage (19.6%) in patients following a GFD [75].

This deficiency can be explained by malabsorption, but GFD may also lead to possible nutrient deficiencies because gluten-free products are usually lower in magnesium, and gluten-free cereals found in nature have a lower magnesium content compared with gluten-containing ones [9].

Furthermore, it has been seen that the resolution of mucosal inflammation may not be sufficient to resolve the shortage of magnesium in celiac patient. The deficiency may also be linked to a reduced intake of this mineral.

Breedon reported that some CD patients need additional magnesium supplement of 200–300 mg/day in the form of magnesium oxide or magnesium chloride, while others can improve magnesium levels through dietary means [76].

#### 3.5.3. Selenium

Selenium deficiency is particularly remarkable because a GFD leads to its absence in cereal foods such as wheat and its derivatives, which are a source of selenium [77]. There are no sufficient literature data to describe a percentage of deficiency investigated through the blood level.

Reduced concentrations of selenium in whole blood, plasma, and leucocytes might develop in several ways. Firstly, GFD might contain a reduced amount of selenium compared with a normal diet, and, secondly, there might be malabsorption of selenium even when the patient is clinically well. Between the extraintestinal symptoms associated with CD, autoimmune thyroid diseases are more evident, underlining that CD-related autoimmune alterations can be modulated not only by gluten but also by various concurrent endogenous (genetic affinity, over-expression of cytokines) and exogenous (environment, nutritional deficiency) factors. The thyroid is particularly sensitive to selenium deficiencies because selenoproteins are significant in biosynthesis and activity of thyroid hormones, while other selenoproteins, including glutathione peroxidase are involved in inhibiting apoptosis. Thus, selenium malabsorption in CD patients can be considered a key factor directly leading to thyroid and intestinal damage [78].

Studies have shown that in celiac patients, selenium supplementation between 120–200 mcg/day is within a safe range. It is important, however, not to exceed the tolerable upper limit of 400 mcg/day for selenium, as this can lead to gastrointestinal upset, hair loss and nerve damage [79].

#### 3.5.4. Vitamin K

Vitamin K deficiency, investigated by markers like PIVKA-II or by prothrombin times, was detected in about 25% of adult patients with CD at the diagnosis, and it seems to return to acceptable levels in almost all patients following a GFD [72,80].

There are limited available data that relate the role of vitamin K and bone health in children and adults with CD. Pazianas et al. examined vitamin K status in children newly diagnosed with CD using prothrombin times as a marker of vitamin K status and found that approximately 35% of children were lacking this marker [72]. However, this may have been an underestimate of the prevalence of vitamin K deficiency as prothrombin time is a very insensitive marker of overall vitamin K status [72]. More sensitive markers of vitamin K status include serum levels of PIVKA-II, which is a vitamin K-dependent protein [80].

In the study carried out by Mager et al., over 25% of children were vitamin K deficient at diagnosis, investigated by PIVKA-II (which is a protein increasing in vitamin K absence), but it resolved in all children after 1 year. This seems to be due in part to improvements in vitamin K intake on the GFD. However, the remaining one-third of children and adolescents continued to have vitamin K intakes considerably lower than the adequate intake on the GFD [80].

Suboptimal dietary intake of vitamin K is common in this population, including when on a GFD. There are no sufficient literature data to recommend a specific dose of supplementation in celiac patient. Therefore, careful consideration should be given to routine supplementation of this nutrient at time of diagnosis of CD.

#### 3.5.5. Niacin, Riboflavin and Thiamin

Deficiency of other micronutrients, like niacin, riboflavin and thiamin have been described in several reviews at the time of diagnosis, although in the literature there is no accurate data on the percentage of celiac patients with such deficiencies [2,16,18]. Deficiencies of niacin and riboflavin may persist after a GFD [2,18]. Regarding thiamin, a study carried out by Shepherd et al. found that the inadequacy of thiamin was more common after GFD implementation than at time of diagnosis [81]. This can be explained by the fact that many gluten-free cereal products do not provide the same levels of thiamin, riboflavin, and/or niacin as enriched wheat flour products. As a result, a GFD that routinely includes gluten-free cereal products could be deficient in one or more of these nutrients, especially if these foods are, in large part, refined and unenriched [82].

Although there is insufficient data in the literature to recommend a dose for supplementation, it is considered useful to carry out control of blood values after diagnosis and after a period of GFD.

## 4. Discussion

It is evident from the analyzed reviews that there emerges an attention towards nutritional deficiencies that occur in celiac patients after an even longer period of GFD with good compliance. The suggested course of action is a half-yearly routine search in patients on LTGFD nutritional deficiencies, such as low levels of folic acid, vitamin B12, vitamin D, calcium, iron, zinc, selenium and magnesium and the need to establish a personalized supplementation plan, following patients over time, to avoid stopping of integration once the values are returned to their normal range, as shown in Table 2.

To help patients reduce deficiencies of minerals (calcium, phosphorus, sodium, potassium, chloride and magnesium) and trace elements (iron, zinc and selenium) it is important to advise them to introduce into their eating habits pseudo-cereals, in which the content of these elements can be twice as high as in other cereals. For example, in teff, iron and calcium contents (11–33 mg/100 g and 100–150 mg/100 g, respectively) are higher than those of wheat, barley, sorghum and rice [18].

It seems important to explain to patients that nutritional education and dietary supplementation should become part of the therapeutic process, which must last a lifetime.

## 5. Conclusions

In conclusion, if correct GFD is not enough, and the blood levels of micronutrients remain low, it is mandatory to start with personalized supplements. In this case, it would be helpful to evaluate the initial blood level to determine the right dosage of supplementation and repeat the examinations to keep under control values.

In any case, there are a lot of unresolved questions regarding the causes and the mechanisms that lead to these nutritional deficiencies. Further studies are absolutely required for the detailed understanding of this topic.

## Figures and Tables

**Table 1:** Reviews on nutrient deficiencies in celiac patients at time of diagnosis and after GFD.

Authors	Type of Study	Country and Year	Results
[2]	Review	Italy, 2010	Common nutrient deficiencies in celiac subjects at diagnosis are: iron, calcium, magnesium, vitamin D, zinc, folate, niacin, vitamin B12, riboflavin, calorie/protein, and fiber. Deficiencies in folate, niacin, and vitamin B12 may occur after LTGFD.
[9]	Review	Italy, 2016	Low levels of fibers, folate, vitamin B12, vitamin D, calcium, iron, zinc and magnesium are common at diagnosis stage. In some subsets of treated celiac disease (CD) patients they can persist.
[16]	Review	USA, 2005	Deficiencies in fiber, iron, calcium, vitamin D, magnesium, zinc, folate, niacin, vitamin B12, and riboflavin can occur at time of diagnosis. Deficiencies in fiber, iron, calcium, vitamin D, and magnesium can persist after following a GFD. Diet and gluten-free products are often low in B vitamins, calcium, vitamin D, iron, zinc, magnesium, and fiber.
[17]	Review	Italy, 2013	Reduced levels of iron, folate, vitamin B12, and vitamin D are common at the time of diagnosis. After GFD low levels of folate, vitamin B12 and vitamin D can persist.
[18]	Review	Italy, 2013	Common deficiencies at diagnosis include: fiber, iron, calcium, vitamin D, magnesium, zinc, folate, niacin, and vitamin B12. Deficiencies of fiber, iron, calcium, vitamin D, magnesium, zinc, folate, niacin, vitamin B12 may persist after following a GFD. Deficiencies of fiber, folate, niacin, vitamin B12, and riboflavin may persist after LTGFD.

**Table 2:** Supplementation of nutrients in generic state deficiency and in celiac patient.

Nutrient	Route of Administration	Dosage and Sources
Vitamin B12	Oral preferable to intramuscular	500 mcg/day ** [24] 1000 mcg orally until the level is normal and then consider daily gluten-free multi vitamin/mineral supplement ** [30] 2000 or 1000 mcg/day, then 1000 mcg/week, then 1000 mcg/month * [29]
Iron	Oral preferable to intravenous	A study on 25 pediatric patients with celiac disease and iron deficiency showed good efficacy of oral administration of iron, (investigated by Bisglycinate Ferrous Chelate) both in patients with gluten-free diet and in those newly diagnosed ** [47] Therapeutic dose in pediatric patients: 3 mg of elementary iron/kg/day. Prophylactic dose in pediatric patients: 2 mg of elementary iron/kg /day until a maximum dosage of 30 mg/day ** [36] Iron supplements (325 mg) 1–3 tablets based on initial ferritin level until iron stores are restored. Consider i.v. iron for severe symptomatic iron deficiency anemia or intolerance of oral iron ** [30] Ferrous sulphate 200 mg 1 or 2/day, (ferrous fumarate, ferrous gluconate) or formulations (iron suspensions) that may also be tolerated better than ferrous sulphate. Oral iron should be continued for 3 months * [46] Therapy should start with a low dose (one tablet/day of any ferrous sulphate commercially available or any other type of iron), and the intake should be constant until the iron deposits are not restored * [40] Intravenous ferric carboxymaltose is a stable complex with the advantage of being non-dextran-containing and a very low immunogenic potential and therefore not predisposed to anaphylactic reactions. Its properties permit the administration of large doses (15 mg/kg; maximum of 1000 mg/infusion) in a single and rapid session (15-min infusion) * [45]
Folic acid	Oral preferable to parenteral	−800 mcg/day ** [24] 1 mg/day of folic acid for 3 months and once diarrhea improves 400–800 mcg/day ** [30]
Vitamin D—Calcium	Oral preferable to parenteral	50.000 U.I./week for 8 weeks, then reduce the dose ** [66] 1000 mg of calcium and 400 U of vitamin D daily ** [67] Calcium: 1000–1500 mg/day in two or more divided intakes of dairy products. If the patient is not able or willing to fulfill the required intake through the diet, calcium supplements can be given. Vitamin D: dose necessary to maintain a blood level of 30 ng/mL ** [68] Vitamin D: 1000 (or more-based serum level) U.I./day or 50.000 U.I. weekly if level is <20 ng/mL. Calcium: recommended intake of calcium, including supplementation, for patients with CD is 1200–1500 mg/day ** [30] -Vitamin D: ≥800 IU/day, at least for 6/12 months of supplementation * [65]

* Therapy in literature in generic state deficiency ** Therapy in literature in celiac patient.

## Supplementary Materials

The following are available online at https://www.mdpi.com/1010-660X/55/7/337/s1, Table S1: Original articles concerning circulating levels and supplementation of micronutrients in celiac patients.
