# Supplementary material for: Micronutrients Dietary Supplementation Advices for Celiac Patients on Long-Term Gluten-Free Diet with Good Compliance: A Review
Source: Medicina (Kaunas). 2019 Jul 3;55(7):337. doi: 10.3390/medicina55070337 (PMC6681258; doi:10.3390/medicina55070337)
Supplement: Supplementary file 1 [file medicina-55-00337-s001.pdf]

Table S1. Original articles concerning circulating levels and supplementation of micronutrients in celiac patients.

First author, year	Population (M/F); age	Time spend on GFD	Primary endpoint	Results
Dahele, 2001	Thirty-nine celiac adults (7M/32F); median age: 48 years (range 22-77 years)	Median 4 months (2-13 months)	PLASMA LEVELS vitamin B12, folate	Sixteen (41%) patients were vitamin B12 deficient (<220 ng/L). Concomitant folate deficiency was present in only 5/16 (31%) of the vitamin B12 patients, suggesting that the subnormal vitamin B12 levels were not secondary to folate deficiency
Dickey, 2008	One hundred celiac adults (29M/71F) (35 newly diagnosed; 24 with persistent gluten atrophy; 41 with recovered villous atrophy); median age: 55 years Two hundred healthy controls	At least 12 months	PLASMA LEVELS Vitamin B12	Both untreated and persistent VA patients have lower vitamin B12 concentrations, there were no significant differences in vitamin B12 concentrations between controls and any of the patient groups.
Hallert, 2002	Thirty celiac adults (12M/18F); median age: 55 years (range, 45–64 years)	Median 10 years (8-12 years)	PLASMA LEVELS Folate, vitamin B6, vitamin B12	Eleven patients (37%) had low plasma vitamin B6 levels; 6 (20%) patients had low plasma folate levels; None had low plasma vitamin B12
Howard, 2002	Two hundred fifty-eight patients celiac adults (26M/232F); median age: 47 years (range 16–80 years).		PLASMA LEVELS Iron, folate	Two hundred forty-seven patients had iron deficiency alone, 10 folate deficiency alone, and one combined iron and folate deficiency. Twelve (4.7%) of the 258 patients with iron and/or folate deficiency who consented to coeliac disease antibody testing had histologically confirmed CD
Hallert, 1981	Forty-eight celiac adults (18M/30F); men median age: 48.1 years (27-70 years), women median age 50.1 years (range 26-82 years)		PLASMA LEVELS	The serum folate levels were low in 40 patients (85%)
Sategna-Guidetti, 2000	Eighty-six celiac adults (22M/64F), men median age: 29 years, (range 19-67 years), 54 pre-menopausal women, median age: 29 years (range 19-51 years) and 10 postmenopausal, median age: 55 years (range 45-67 years)	1 year	PLASMA LEVELS	At time of diagnosis, approximately 50% of patients had anemia, caused by isolated deficiencies of iron and/or folic acid. A 1-year GFD led to a significant improvement in bone mineral density, bone metabolism and nutrition, except for folic acid, albumin and pre-albumin serum levels which persisted as abnormal in patients with obdurate mucosal impairment. Intestinal biopsy which showed a mucosal recovery in only 57%.
Larussa, 2012	Seventy celiac adults (13M/57F); median age: 40.5 years (range 20-68 years)	At least 2 years	PLASMA LEVELS Calcium	No patient showed low levels of serum calcium

Larussa, 2017	Sixty-four celiac adults (18M/46F) median age: 36 years (range 18–69 years)	At least 2 years	PLASMA LEVELS Calcium, vitamin D	Serum calcium and vitamin D were normal
Rujner, 2004	Forty-one pediatric celiac patients (12M/29F), boys median age 12.8 years (range 5.9-16.7 years), females mean age 13.6 years (range 5.9–18.3 years); 28 untreated patients and 8 healthy controls	Median 11 years (2.7-17.3 years)	PLASMA LEVELS Magnesium	Tissue Mg deficiency in 1/5 of the examined patients with CD. Mg deficiency was clinically symptom-less and occurred with a similar frequency in coeliac patients treated with a GFD, with subclinical CD, and in control group, and was equally frequent in males and females
Hallert, 2009	Sixty-five celiac patients (24M/41F), aged 45–64 years	Median 15.5 years (8–29 years)	SUPPLEMENTATION B vitamin supplementation (daily dose of 0.8 mg folic acid,0.5 mg cyanocobalamin and 3 mg pyridoxine or placebo for 6 months)	Improvement of psychological well-being, and a significant return to normal vitamin B12 values with reduction of homocysteine values in treatment group
Muzzo, 2000	Nineteen celiac children (3M/16F), age 6-15 years, 19 healthy controls	At least 2 years	SUPPLEMENTATION 1000 mg of calcium and 400 U of vitamin D daily for 24 months	Celiac patients had mean calcium intakes of 739 mg per day, that increased to 1444 mg per day after nutritional supplementation, with 84 and 74% of compliance during the first and second years of supplementation.

References

Dahele, A.; Ghosh, S. Vitamin B12 Deficiency in Untreated Celiac Disease. *Am. J. Gastroenterol.* **2001**, *96* (3), 745–750.

Dickey, W.; Ward, M.; Whittle, C. R.; Kelly, M. T.; Pentieva, K.; Horigan, G.; Patton, S.; McNulty, H. Homocysteine and Related B-Vitamin Status in Coeliac Disease: Effects of Gluten Exclusion and Histological Recovery. *Scand. J. Gastroenterol.* **2008**, *43* (6), 682–688.

Hallert, C.; Grant, C.; Grehn, S.; Grännö, C.; Hultén, S.; Midhagen, G.; Ström, M.; Svensson, H.; Valdimarsson, T. Evidence of Poor Vitamin Status in Coeliac Patients on a Gluten-Free Diet for 10 Years. *Aliment. Pharmacol. Ther.* **2002**, *16* (7), 1333–1339.

Howard, M. R.; Turnbull, A. J.; Morley, P.; Hollier, P.; Webb, R.; Clarke, A. A Prospective Study of the Prevalence of Undiagnosed Coeliac Disease in Laboratory Defined Iron and Folate Deficiency. *J. Clin. Pathol.* **2002**, *55* (10), 754–757.

Hallert, C.; Tobiasson, P.; Walan, A. Serum Folate Determinations in Tracing Adult Coeliacs. *Scand. J. Gastroenterol.* **1981**, *16* (2), 263–267.

Sategna-Guidetti, C.; Grosso, S. B.; Grosso, S.; Mengozzi, G.; Aimo, G.; Zaccaria, T.; Di Stefano, M.; Isaia, G. C. The Effects of 1-Year Gluten Withdrawal on Bone Mass, Bone Metabolism and Nutritional Status in Newly-Diagnosed Adult Coeliac Disease Patients. *Aliment. Pharmacol. Ther.* **2000**, *14* (1), 35–43.

Larussa, T.; Suraci, E.; Nazionale, I.; Leone, I.; Montalcini, T.; Abenavoli, L.; Imeneo, M.; Pujia, A.; Luzza, F. No Evidence of Circulating Autoantibodies against Osteoprotegerin in Patients with Celiac Disease. *World J. Gastroenterol.* **2012**, *18* (14), 1622–1627.

Larussa, T.; Suraci, E.; Imeneo, M.; Marasco, R.; Luzza, F. Normal Bone Mineral Density Associates with Duodenal Mucosa Healing in Adult Patients with Celiac Disease on a Gluten-Free Diet. *Nutrients* **2017**, *9* (2), 98.

Rujner, J.; Socha, J.; Syczewska, M.; Wojtasik, A.; Kunachowicz, H.; Stolarczyk, A. Magnesium Status in Children and Adolescents with Coeliac Disease without Malabsorption Symptoms. *Clin. Nutr.* **2004**, *23* (5), 1074–1079.

Hallert, C.; Svensson, M.; Tholstrup, J.; Hultberg, B. Clinical Trial: B Vitamins Improve Health in Patients with Coeliac Disease Living on a Gluten-Free Diet. *Aliment. Pharmacol. Ther.* **2009**, *29* (8), 811–816.

Muzzo, S.; Burrows, R.; Burgueño, M.; Ríos, G.; Bergenfreid, C.; Chavez, E.; Leiva, L. Effect of Calcium and Vitamin D Supplementation on Bone Mineral Density of Celiac Children. *Nutr. Res.* **2000**, *20* (9), 1241–1247.
